# Hidden Signals—The History and Methods of Heart Rate Variability

**DOI:** 10.3389/fpubh.2017.00265

**Published:** 2017-10-16

**Authors:** Gernot Ernst

**Affiliations:** ^1^Anesthesiology, Pain and Palliative Care Section, Kongsberg Hospital, Vestre Viken Hospital Trust, Kongsberg, Norway

**Keywords:** heart rate variability, Holter monitoring, time domain, frequency domain, systems science, complexity theory

## Abstract

The understanding of heart rate variability (HRV) has increased parallel with the development of modern physiology. Discovered probably first in 1847 by Ludwig, clinical applications evolved in the second part of the twentieth century. Today HRV is mostly used in cardiology and research settings. In general, HRV can be measured over shorter (e.g., 5–10 min) or longer (12 or 24 h) periods. Since 1996, most measurements and calculations are made according to the standard of the Task Force of the European Society of Cardiology and the North American Society of Pacing and Electrophysiology. As the first step, the series of times between successive R-peaks in the ECG are in milliseconds. It is crucial, however, to identify and remove extrasystoles and artifacts according to standard protocols. The series of QRS distances between successive heartbeats can be analyzed with simple or more sophisticated algorithms, beginning with standard deviation (SDNN) or by the square root of the mean of the sum of squares of differences between adjacent normal RR (rMSSD). Short-term HRV is frequently analyzed with the help of a non-parametric fast Fourier transformation quantifying the different frequency bands during the measurement period. In the last decades, various non-linear algorithms have been presented, such as different entropy and fractal measures or wavelet analysis. Although most of them have a strong theoretical foundation, their clinical relevance is still debated.

## Introduction

Heartbeat varies over time. This has been observed early in medicine. Variations and patterns of heart beat have been associated with pathological conditions already 2,000 years ago (1). However, first in the last 100 years, conceptual ideas evolved, and understanding of involved mechanisms increased, in particular since 1996 when a standard was established and parameters defined (2).

The increasing interest in heart rate variability (HRV) can partially be explained by the feasibility of the method. Data can in principle be obtained by a simple one-channel ECG or even a pulse watch; data are processed by user-friendly programs. In reality, the issue is more complicated {(3) #2021} {(4) #2317} {(5) #2320}. Whether pulse watch-generated HRV calculations can be used is still a matter of debate {(6) #2326} {(7, 8) #2022} {(9) #2319}. Automated recognition of R-peaks is prone to errors {(10) #2323} and manual editing is still the gold standard, which impairs clinical use. No overall accepted normal values exist. In the beginning, HRV was first usually calculated based on 24-h recordings. Eventually, new algorithms were introduced (explained below) and clinical studies supported the use of short-term measurements. HRV has thus changed to an apparently simple point-of-care method obtained within 2–10 min with potential clinical value for the patients regarding risk stratification, individual therapeutic strategies, and even therapeutically in the form of HRV-biofeedback.

This review intends to give an overview of the developments of HRV in the last decades. It is basically descriptive. In earlier work (11), an extensive literature search was conducted, based mainly on the simple keyword “Heart Rate Variability” in the US National Library of Science (PubMed) and consecutive search in the reference lists of the identified articles. This review extends and updates this work although only the most central publications, chosen by the author will be discussed, for the sake of clarity.

Therefore, in this review, only a brief history of HRV will be presented. In the second part, the methods of signal measurement will be introduced. Most important algorithms for HRV analysis will be explained, but algorithms (still) not being used in clinical research or practice will not be mentioned. In addition, some possible confounding mechanisms of importance will be reported. Finally, a brief perspective of HRV for the future will be offered.

## History

Pulse diagnosis has been early a part of ancient medicine and descriptions include its variation over timeWestern medical historians usually quote Galen as one of the first analyzing pulse patterns in human patients. Pulse diagnosis was, however, an important part of ancient Chinese and Indian medicine, too. In China, pulse diagnosis was investigated as early as between 800 and 200 BCE. For instance, in Chinese Medicine, Bian Que (扁鹊, about 500 BCE, also known as Qin Yueren, 秦 越人), living about one generation before Hippocrates described the “four diagnostic methods” of Traditional Chinese Medicine, in particular tongue and pulse diagnostics. All these forms of historical pulse analysis described patterns qualitatively. Quantitative measurements were first possible after the introduction of exact time measuring devices.

Variations of arterial blood pressure during the respiratory cycle was observed again in the eighteenth century, probably first of Stephen Hales. His observation of HRV was based on conducting measurements of blood pressure in some animal species (mostly dogs) by inserting fine cannulas into arteries and measuring the height to which the column of blood rose (12). Carl Ludwig (1816–1895) described a link between heartbeat fluctuations and respiration [respiratory sinus arrhythmia (RSA)] when investigating the frequency and pulse wave in dogs using a special instrument (“kymograph”) (13). One of the founders of experimental psychology, Wilhelm Wundt (1832–1920), made similar observations and introduced the notion of using physiological measures to investigated psychological mechanisms.

The French physiologist, Claude Bernard (1813–1878), introduced the term “milieu intérieur,” a basic principle to homeostasis. This internal environment is “constituted, in particular, by the fluids circulating in the body.” The American physiologist, Walter Bradford Cannon (1871–1945), expanded Bernards concept of homeostasis, beyond others by the two claims that the regulating system determining the state of the homeostasis consists of several connected subsystems. According to Cannon homeostasis is a consequence of self-organizing systems (termed self-government by Cannon). An important paradigm in HRV is based on Bernard’s and Cannon’s notion. Stable homeostasis is according to this concept connected to increased variability of HRV (14).

The classical model of autonomic control describes a balance between parasympathetic and sympathetic activation. It was also proposed by Cannon (15) and later expanded by Langley (16) who divided the autonomic outflows between sympathetic and parasympathetic elements, a division used until today. Cannon associated also increased activity in the sympathetic system with the evolutionary notion of “fight and flight.” In his seminal book, Langley erroneously defined the ANS as a purely visceral motor system, mediating the consequences of central nervous states to the periphery [today we know that 80% of vagal fibers are in reality afferent, providing important information to the brain regarding the state of the visceral organs (17)]. Hering described the functional relation between the amplitude of RSA and the vagal tone in 1910 (18). His son provided experimental data describing the baroreceptor reflex more exactly in 1927 (19).

Some years later, Adrian et al. published for the first time the behavior of the sympathetic nervous system in anesthetized rabbits and cats (20). At the same time, Maltzberg observed the association between cardiac disease and major depression, at this time termed “involution melancholia” (21), an association leading to important research in the last decades.

In 1965 investigations of the HRV of fetal ECGs revealed diminished variability after contractions when the fetus was distressed (22). This principle is still a cornerstone in monitoring fetus under labor. In cardiology, the relationship between the nervous system status and HRV was described by Wolf (23), 2 years after Valbona et al. described HRV changes in patients with serious brain damage (24).

Katona and Jih (25) introduced a non-invasive approach to measuring cardiac parasympathetic control in anesthetized dogs where they were able to control respiration rate. They introduced the notion that the magnitude of sinus arrhythmia is associated with changes in the vagal tone; assuming a linear association between vagal efferent activity and the change of heart period, and that during inspiration the cardiac vagal input is inhibited. Akselrod et al. applied power spectrum analysis of short-term HRV in an animal model, showing the association between different frequency ranges and the sympathetic and parasympathetic activity (26).

At this time, portable ECG-measurements became more frequent. Until then, HRV was mainly determined by measuring RR-distances with a ruler. Eventually, electrical circuits identifying the peak of R-waves and the time of the intervals with an accuracy of milliseconds were developed. First investigations of HRV were based on 24-h recordings by Holter monitoring. This changed when Axelrod started to analyze the frequency domain of HRV also in humans by using short-term HRV of 10 min or less (27). Earlier, spectral analysis methods were utilized in some study, investigating driver fatigue {(28) #2332}, the effect of aging on HRV {(29) #2333}, or in hypertension {(30) #2334}. Of particular importance at this time was also the increasing interest in non-linear phenomena. In particular Goldberger, the founder of the prominent website “Physionet,” began to focus more on non-linear algorithms (31–33). Looking closer on his articles shows relevant influences: he quotes May’s important article about evolutionary models (34) and Haken’s (35), and Shaw’s articles about chaos theory and strange attractors (36). Hermann Haken, physicist conducted research on self-organizing systems and founded at the end of 1960s synergetics, an interdisciplinary science investigating the formation and self-organization of patterns and structures in open systems far from equilibrium—characteristic for most physiological systems {(37) #2327}. Robert May introduced the use of models to test stability and fragility of systems {(38) #2328}. Robert Shaw was one of the pioneers of chaos theory at this time.

The breakthrough of HRV in cardiology occurred when the association between SDNN (explanation, see below) and the mortality after acute myocardial infarction was discovered. Probably, the first observation was made public by Australian group 1978, describing an association between sinus arrhythmia and survival after acute myocardial infarction {(39) #2329}. A landmark study of Kleiger et al. {(40) #1561} and several important cardiologic HRV studies followed, e.g., Ref. (41–44), frequently combining traditional cardiological measures with HRV. Bigger’s introduction of short-term measures (45) and Kleiger’s study were significant reasons to form the joint Task Force of the European Society of Cardiology and the North American Society of Pacing and Electrophysiology (2). The Task Force proposed minimal technical requirements, definitions, standardized the areas of Power bands in frequency domain and offered recommendations for clinical research and patient examinations. This article is still the most frequently cited HRV paper. Nearly every study after 1996 is based on this standard, and no major revision appeared until recently—the presentation of currently accepted linear measures is comprehensive, and the clinical signification of the non-linear parameters is still unclear. A recent joint position statement of the European Society of Cardiology and the European Heart Rhythm Association stated a lack of communication between mathematicians and engineers developing new algorithms and clinicians. It recommends, however, the combined use of linear and non-linear measures (46). A recent study provided reference values obtained by healthy individuals (7, 8), with limited relevance because they were recorded with Holter monitoring 24 h and are, therefore, not applicable for short-term measurements. The study was also criticized because of inconsistencies and unrealistic values, beyond others (3).

Already more than one century ago scientists observed and proposed associations between imbalances of the ANS and (pathological) mental states. Notions included that dysfunctional mental states might be associated with excessive vagal outflow (47), with imbalances between the sympathetic and parasympathetic system (48), or with excessive sympathetic outflow (49). Already Lacey and Lacey reported personality traits associated with greater HRV (50). Early work of Porges and Raskin showed mental state associations with HRV (51). This notion was later extended and elaborated by Porges (Polyvagal Theory) and Thayer (Neurovisceral Integration Model) (52–55).

Today, HRV has been used in more than 2,000 clinical trials and has been mentioned in more than 14,000 articles (46). It is used as an algorithm in sports watches and frequently appears in new Apps in electronic devices, mostly for health or training purposes (7, 8). The clinical use, however, is still invariant.

Probably the most relevant use of HRV in clinical practice is risk stratification. Several studies have shown clear associations between decreased HRV and the risk of sudden cardiac death (56–58) and the value of using HRV has been recognized (58–60). In some centers, HRV, together with other variables is used to identify patients at high risk for sudden cardiac death (61). This has consequences for treatment because the identified individuals received Automated Implantable Cardioverter-Defibrillators, an expensive, but a highly effective method. HRV is also established in the identification of cardiac autonomic neuropathy caused by diabetes and part of standardized examination protocols (62, 63). An emerging field is the use of HRV to predict systemic infections in critical care medicine. However, HRV is only utilized in some hospitals, and more often still not implemented in clinical practice (64, 65).

Based on the mentioned models and concepts above, HRV is also increasingly used in psychological research. The general hypothesis there is that higher levels of HRV parameters associated with activity in the parasympathetic system are also associated with better adaptivity to perturbations and better stress response. A recent meta-analysis confirmed this hypothesis, showing significant associations, although the absolute differences were small. Interestingly not only parasympathetic but also higher general HRV parameters were related to greater adaptivity (66). As an example, HRV has been used as a method in anxiety research. According to the neurovisceral model, anxiety disorders can be characterized by a breakdown of the inhibitory processes of the central autonomic network (67). This disinhibition is permanently linked to the continual state of excessive worry and mirrored by the decreased activity of the parasympathetic system. Several studies have investigated individuals with different kinds of anxiety disorders and have supported this notion in general anxiety (68), various forms of panic disorder (69), social anxiety (70), stress-associated anxiety (71), and trait anxiety (72). A closer look at these studies also shows the problems—e.g., in an experimental study looking on correlations between electric skin conductance, startle blink reflex and resting HRV (rMSSD) during conditioned fear inhibition and extinction. Higher rMSSD was associated with pronounced fear inhibition and extinction (indexed with startle blink potentiation), but the effect is most pronounced at the group level, and the scatter plot shows rather a point cloud instead of a clear regression line (73).

The newer history of HRV research is closely associated with the history of complexity research. As already mentioned, Ary Goldberger was inspired by publications of beyond others von Haken. He is one of the European representatives of a research tradition trying to understand systems. A system is regarded as a set of different parts (or subsystems) connected through positive and negative feedback circles. The fundamental notion of complexity science is that the whole system has more properties as the sum of properties of its parts. In other words, if you analyze the parts of the system separately, and add all results, there will be properties which cannot be explained out of this. Another term for this approach is non-linear science. Linear systems can be described by an addition of the equations describing its parts. If the subsystems interact, the system behaves non-linear and its behavior cannot be predicted by analyzing its parts. A set of equations characterizing a non-linear system can usually neither be solved with analytical mathematical methods {(74) #2335}.

Non-linear systems behave different compared to linear systems {(75) #2352}. Key notions are robustness and fragility. System robustness is often defined as the quality of a biological system or network to maintain its components, structure, and function despite both external changes and endogenous fluctuations {(76) #2337} {(77) #2338}. Fragility is connected to robustness. A property of complex systems is a conservation of sensitivity. When robustness is improved in one area, it leads to increased fragility in another {(78) #2339}. Complex systems are, therefore, robust, yet fragile by cascading failures initiated by tiny perturbations which may lead to a complete breakdown, or to a fundamental system change, termed emergence {(79) #2340}. Essential tools to study complexity are mathematical models and time-series analysis. HRV is the most used time-series analysis in medicine. The complexity paradigm has been explicitly used of Thayer and Lane in their neurovisceral model (55). The study of HRV has been influenced by dynamical systems theory, the study of fractal systems and chaos theory. It was also influenced by notions of self-organizing systems, network theories, and by modeling methods {(11) #1498}.

## Methods

Investigating HRV needs a three-step approach. First, a condition should be defined where the measuring of heart rate signals and its variability gives relevant information. For the second, it is important to detect the signal as adequate as possible, to identify potential artifacts and manage them and at the end to obtain a time-series in milliseconds between the heartbeats which can be analyzed. The third step consists of different forms of analysis which again return various parameters to be used to analyze the state of the system.

Preparing a measurement of HRV should involve answers to several questions. The length of measurement is relevant for the kind of parameters of interest. When the focus is on basic parameters, a measurement period of 5 min or even less might be enough. When long-term fluctuations are relevant, a longer measurement period is necessary. Several non-linear parameters do also need a longer measurement period than 5 min. Details are given in Table 1. Recently, ultra-short-term analysis has been proposed for some parameters (80). According to these reports, the time domain measure root Mean Sum of Squared Distances (rMSSD) and the frequency domain measure High Frequency Power (HF, both explained in the next sections) can be reliably measured in time-series of 10–30 s.

**Table 1:** Measurement period and parameters.

Measurement period	Possible parameters
6 s to 2 min	SDNN [Dekker et al. (81) and Carnethon et al. (82)]
5 min	In addition: rMSSD, HF, LF, LF/HF
10 min	In addition: VLF, approximate entropy
hours	In addition: fractal measures
24 h	In addition: ULF

Most algorithms for the analysis of short-term HRV require stationarity of the heart rhythm. The heart rhythm should not increase or decrease during the measurement period. An exact rule of stationarity would demand that the distribution of a time-series is invariant over time. A weaker rule demands only that mean and covariance are stable. When in time-series trends are occurring, they can probably distort the parameter calculation (45). Stationarity in measurement protocols is usually obtained by demanding a resting period for the individuals at least 5, but usually 10 min. In the case of measurements during tests (e.g., physical movement, stress tests) algorithms not needing stationarity should be considered.

Another precondition is of course that the heart rhythm is feasible for HRV analysis. Although some research groups have used HRV analysis in atrial fibrillation (AF) {(83) #1868} {(84) #1719} {(85) #2341}, in most cases individuals with AF have to be excluded. The same applies for participants with a high number of ectopic beats, with exception when heart rate turbulence will be analyzed, where ectopic beats are needed. Individuals with more than 20–30% ectopic beats are usually not feasible for HRV analysis {(86) #2343}. Before HRV parameters can be calculated, preprocessing of the raw data is necessary. Artifacts have to be removed, and ectopic beats have to be identified and handled. Several computer-based algorithms provide automatic identification and managing of ectopic beats, but most protocols include a manual review of the ECG signal {(86) #2343}. A typical way of management is to replace the distances between the QRS complex before and after the ectopic beat by the distance between these two QRS-complexes divided by two {(2) #1505}.

The sampling rate is an important issue. If the sampling rate of the signal is under a certain threshold, the calculated parameters might be distorted. Wittling showed that a sample rate below 256 Hz can already cause significant distortion with the example of a patient investigated after myocardial infarction (87) p 151. The Task Force recommends a sampling rate between at least 250 and 500 Hz. A lower sampling rate is only acceptable if appropriate interpolation algorithms are used, but not lower than 100 Hz (2). A recent exploration described stable measures at sampling rates of 125 Hz or lower (88).

In the last years, heart rate has been increasingly measured by photoplethysmography (PPG), as implemented in newer smart watches {(9) #2319}. For instance, the pulse watch Polar RS800cx, using an electrode belt and PPG measured with a finger cuff, compared with ECG showed moderate to excellent agreement levels. However, some values (LF and HF) had a lower correlation {(89) #2321}. Mobile phone technology showed excellent similarity between ECG signals and finger color changes taped with the camera lens, and the flash turned on {(90) #2324} {(91) #2322}. For instance, SDNN measured by ECG was 92.2 ± 5.3 and 92.3 ± 5.9 by the mobile phone in one study {(91) #2322}. These results seem promising, and this method has been used in some studies, e.g., {(92) #2325}. A recent review concluded that Pulse Rate Variability with PPG seems to work acceptable in healthy younger persons at rest, but not in movement or under stress conditions {(6) #2326}. Application of pulse watches with breast belts or PPG can only be recommended, if the particular equipment is first validated with a traditional ECG approach.

Several factors influence the measurement results. HRV results are clearly sex and age dependent. Also sleep, physical exercise, fasting, and position might distort HRV parameters. An overview is given in Table 2. A comprehensive overview is given in Ref. (11), chapter 4. One major problem regards reference values. Some reference values have been provided by the Task Force {(2) #1505}. In a review, 44 studies with together 21,438 participants were pooled and the results were considerably different {(4) #932}. A recent study provided reference values for 24-h recordings {(7, 8) #2020}, but received massive critic for inconsistencies and the methodological approach {(3) #2021}. Beyond others heterogeneity of study populations, measurement conditions (e.g., stressed or relaxed participants), or time of the measurement can have profound effect. Studies have, therefore, usually control groups instead of relating to reference values. On the other hand, some parameter values, such as SDNN < 50 ms are generally accepted as pathological {(3) #2021}.

**Table 2:** Physiological factors on heart rate variability (HRV).

Factor	Effect	Reference
Sex	Most parameters are lower in women	Stein et al. (93) and Bonnemeier et al. (94)
Age	Most HRV parameters decrease with age, except ULF	Bigger et al. (95) and Stein et al. (93)
Weight	Anorexia nervosa: frequency domain↓. Increased BMI: total power (TP)↓. Weight loss >10%: HF↑	Rechlin et al. (96), Poirier et al. (97), and Kimura et al. (98)
Food intake	Few studies. Eating a meal had no influence. Dietary restriction: HF↑, LF↓	Ambarish et al. (99) and Vogele et al. (100)
Ethnicity	Problematic factor. One study showed lower HRV in Afro Americans compared to Caucasians but did not control social class	Choi et al. (101)
Circadian effects	SDNN↑ at night in one study. Most parameters decreased at night	Viola et al. (102) and Bonnemeier et al. (94)
Sleep	REM sleep: TP, VLF, LF↑, LF ↓. Non-REM sleep: TP, VLF, LF ↓, LF↑. In light sleep, SDNN, LF and LF/HF values are similar to wakefulness. Sleep deprivation: LF↑, conflicting results	Busek et al. (103), Zhong et al. (104), Chung et al. (105), Kesek et al. (106), and Ernst (11)
Regular exercise	SDNN, TP, HF, fractal dimension↑	Nakamura et al. (107), Levy et al. (108), and Pardo et al. (109)

Different drugs might influence HRV parameters, but often the evidence is conflicting. Beta blockers are mentioned most frequently (2), but recently a negative study was published (110). In most studies, individuals taking beta blockers are excluded, analyzed separately or included into the statistical model. An overview of some drugs and its effects on HRV is given in Table 3. Different antidepressive drugs frequently showed effects on various HRV parameters. Amitryptilin and Doxepin, taken in a period of 2 weeks was associated with general decreased frequency domain parameters (111), the effect of tricyclic antidepressants, selective serotonin reuptake inhibitors and other antidepressants was confirmed in a larger study (112) Also here, a comprehensive overview can be found in Ref. (11), chapter 4.

**Table 3:** Effects of drugs on heart rate variability.

Drug	Effect	Reference
Angiotensin II receptor antagonists	Increase of all time and frequency domain parameters	Petretta et al. (113)
ACE inhibitors	Increased total power (TP), HF, LF, ULF, VLF, and SDNN	Binkley et al. (114) and Bonaduce et al. (115)
Beta blockers	TP, HF, LF, and VLF↑, rMSSD, pNN50, SDNN and HF↑, in another study no effect	Pousset et al. (116), van den Berg et al. (84), Lin et al. (117), and Ernst et al. (110)
Antidepressiva	SDNN and frequency domain parameters decreased or unchanged	Rechlin (118), Rechlin et al. (111), Bar et al. (119), Straneva-Meuse et al. (120), and Licht et al. (112)
Caffeine	Increase of SDNN, rMSSD, frequency domain parameters, approximate entropy, detrended fluctuation analysis, or no change	Yeragani et al. (121), Rauh et al. (122), Richardson et al. (123), and Karapetian et al. (124)
Sedatives (midazolam, propofol, thiopental)	Decreased HF and LF	Galletly et al. (125), Michaloudis et al. (126), and Riznyk et al. (127)
Metformin	Increased TP, and HF, decrease in LF and LF/HF ratio	Manzella et al. (128)
Omega-3 fatty acids	Increased SDNN, rMSSD, HF, VLF, or no change	Mozaffarian et al. (129), Mozaffarian et al. (130), and Xin et al. (131)
Digoxin	Increased HF, LF, and rMSSD	Krum et al. (132)

## Algorithms

### Linear Algorithms

#### Time Domain

Time domain analysis measures the variation of the intervals between consecutive normal cardiac cycles. The SD of NN intervals (SDNN) is the most frequently used HRV parameter, formally the SD of all normal (“NN”) QRS distances. It correlates with total power (TP), often *r* > 0.9 (87) {(133) #2347}. Since TP is adjusted to the variance of the analyzed time-series within the particular time frame, this correlation is not surprising. The SD of the average NN intervals (SDANN), usually calculated over 5-min periods, needs longer measuring periods and cannot be applied in short-term HRV measures. pNN50 and rMSSD can be used both in short-term and long-term measurements. NN50 is the number of pairs of successive NNs that differ by more than 50 ms, pNN50, the proportion of NN50 divided by total number of NNs over (normally) a 24h-recording {(2) #1505} and is often interpreted as a proxy for cardiac parasympathetic activity {(134) #2345}. rMSSD stands for the square root of the mean squared differences of successive NN intervals (2, 135). Some reference values for time domain parameters are presented in Table 4. Importantly, time domain parameters depend on the length of the recording time. Longer periods generate more variability. Studies can, therefore, only be compared when they use the same measurement period {(136) #2350}.

**Table 4:** Reference values for some heart rate variability values provided by the Task Force and Nunan {(4) #2317} {(2) #1505}.

	Task force	Nunan (4)
SDNN (ms)	141 ± 39	50 ± 16
rMSSD (ms)	27 ± 12	42 ± 15
LF (ms^2^)	1,170 ± 416	519 ± 291
HF (ms^2^)	975 ± 203	657 ± 777
LF/HF	1.5 ± 2.0	2.8 ± 2.6

### Geometric Methods

Geometric methods are obtained from sequences of NN intervals. Several algorithms are described as geometric methods, such as the 24-h histogram, the HRV triangular index, the triangular interpolation of NN interval histograms, and the Poincaré-plot.

The *triangular index (TI)* constructs a triangle with the major peak of the histogram, its baseline width corresponding to the amount of RR interval variability, and its height corresponding to the total number of all RR intervals (137). It is based on the density distribution (the number of all NN intervals) divided by the maximum of the density distribution. TI uses time-series of NN intervals on a discrete scale, and the parameter is calculated by the total number of NN intervals divided through the number of NN intervals in the modal bin and dependent on the length of the bin, with other words on the precision of the discrete scale of measurement [Task Force 1996]. It has been used more frequently in the last years, e.g., in Ref. (138, 139).

The *Poincaré-plot* is constructed with pairs of following R–R intervals assumed implicitly that the current one significantly determines the next R–R interval. Under physiological conditions, the difference between the first and following QRS-intervals increases, but less under pathological conditions (135). Poincaré plots can be approached qualitatively by describing their different shapes (140) but they can also be measured by the SD12 index which is based on the length of the axis of a circle having its center at the average RR interval and being related to the plot itself (141). Its additional value to other linear domain parameters is limited since SD1 correlates closely to rMSSD and SD2 to SDNN (142).

#### Frequency Domain

The frequency domain (power spectral density) analysis in humans was introduced by Axelrod et al. (27). It describes the periodic oscillations in different frequencies of the heart rate signal, and quantifies the amount of different frequency bands (137). During preprocessing, the RR intervals have to be resampled to transform it to a real time-series, usually at 4 Hz to capture oscillations up to 2 Hz according to the Nyquist theorem {(143) #2349}. Most frequently, frequency domain is calculated non-parametrically with the fast Fourier transformation (FFT). Parametric methods in the discrete Fourier transformation are more complex and dependent on the used model. The investigated time-series has to be stationary; therefore, it cannot be applicated in patients with fast changing heart rates under the measurement period. Under certain circumstances FFT fails to find structures which can be found with, e.g., wavelet analysis (144).

Usual parameters include TP, VLF (very low frequency, <0.003–0.04 Hz), LF (low-frequency power, 0.04–0.15 Hz), HF (high frequency power, 0.15–0.4 Hz). A frequently used ratio is LF/HF. Frequency domain parameters can be applicated both in short- and long-term measurements, but not ULF (ultra low frequency, <0.003 Hz), which only can be used in Holter monitoring.

HF is frequently interpreted as a marker of the PNS and is influenced by the respiratory rate (135). It is to a certain degree the same as the RSA (45) and correlates with it (145). Parasympathetic regulation of the heart has a fast response after about 0.5 s and returns to baseline within 1 s (67).

LF is modulated both by the activity of the sympathetic and parasympathetic system. A high LF power is often explained as result of high sympathetic activity (mental, physical stress, sympathomimetic pharmacologic agents). Sympathetic input leads to changes in heart rate, however, more slowly as after parasympathetic input, with a peak after about 4 s and return to baseline after about 20 s (146). The LF/HF ratio mirrors the general sympathetic/parasympathetic balance and returns usually in rest a value between 1 and 2. VLF is a general proxy for physical activity and might mirror also sympathetic activity, but the causality is debated (135). Increased inflammatory parameters like CRP, Il-6, and WBC are correlated with low VLF (147). Some reference values for frequency domain parameters are presented in Table 4.

### Non-Linear Algorithms

The difference between “linear” and “non-linear” methods in HRV is not as straightforward as in the general definition mentioned above. Principally, frequency domain analysis is based on already established patterns. In Fourier transformations, the presumed frame is a sinusoidal wave and in wavelet analysis predefined wavelet function. Both patterns are in principle non-linear, but the methods remain linear because in Fourier transformations the sine waves are added, same as in wavelet analysis the different wavelets. By contrast, non-linear methods are not based on prespecified structures but analyze temporal similarities in the signals. Entropy is frequently, but not entirely, described as a measure for regularity of the signals, whereas fractal methods investigate self-similarities within signals.

### Entropy

An influential algorithm in HRV at the beginning of the 1990s was approximate entropy (ApEN) (148). It was first introduced in 1991 (149) and evaluates data sets for repeating structures and for the probability that other time periods in the data set with the same length of runs (*m*), tolerance (*r*), and length of RR intervals (*n*) have the same structures. ApEN returns a number between 0 and around 1. In normal adults, ApEN is around 1. Lower numbers of ApEn indicate higher regularity, higher values less patterns and low uniformity in the data set. ApEN can be used reliably down to 1,000 data points making it feasible for short-term-HRV of 20 min (148). ApEN has been used successfully in such different fields in endocrinology (secretion of ACTH and cortisol in patients with major depressive disorders) (150), HRV behavior in patients with a combination of unstable angina pectoris and depression (151), respiration patterns in panic disorders (152), or HRV of adolescents treated with anti-depressant drugs (153). ApEN and other similar tools are superior to detect unknown relations between seemingly unconnected systems. In one study investigating patients with cachexia due to COPD, they had in contrast to non-cachectic patients with similar disease and healthy controls an absent circadian rhythm of circulating leptin (154). A major problem of ApEN is probably a lack of internal consistency. Therefore, as alternative a different algorithm, termed “sample entropy” (SampEn) has been introduced (155). Similarly, it calculates the probability of identifying specific patterns in a short time-series and is defined as the negative natural logarithm of an estimate for predictability in finding specific matches in a short time-series {(155) #1566}. To set the exactness of pattern recognition, the length (*m*) of the subseries and the tolerance (*r*) for the patterns has to be predefined. It returns results between 0 and around 2, 0 represents, e.g., a sinus curve and a result near 2 complete chaos. SampEn needs far fewer data points compared to ApEN, and it can be applicated in time-series between 200 to 250 data points (156, 157). Several other entropy algorithms have been proposed, like Lempel Ziv entropy (158), Multiscale entropy (159), fuzzy entropy (160), or Renyi entropy (161). All have been used in clinical studies, but their significance is still unclear.

### Fractal Analysis

The notion of fractality has been originally introduced and applied by Benoit Mandelbrot on spatial self-similarities in graphical plots of non-linear deterministic iterations (162). Used for heart rate time-series, it refers not to spatial, but to temporal self-similarities over a range of scales {(163) #2351}. A normal series of RR intervals is fractal-like and shows a scale-free 1/f fluctuation typical for self-organizing systems behaving between uncorrelated randomness and highly predictable behavior {(164) #1569} {(165) #1747}.

Detrended fluctuation analysis (DFA) determines the statistical self-affinity of a signal. When used to analyze heart rates, it yields to separate scaling regions, a short-term scaling exponent and a long-term scaling exponent. Peng et al. presented the short-term scaling exponent (also termed α1) calculated by DFA first in genetical data (166) and in the following year also in HRV (167). Its great advantage is that it can be used for non-stationary data from time-series and correlates with the randomness in the heart rate time-series, the lowest values (~0.5) ressembles an entirely random series; high values (1.5) signify a time-series being completely correlated (141). It has been used to predict cardiac mortality in different patient populations (165, 168). Unfortunately, it needs at least 1,000 beats and has, therefore, been used more frequently in Holter monitoring studies.

Other proposed algorithms include coarse grained spectral analysis (169), the Fano factor (170), dispersional analysis (170, 171), fractal dimension (172), correlation dimension (173), or the Largest Lyapunov Exponent (174). Their clinical value is still unclear.

### Other Algorithms

*Heart Rate Turbulence* is normally not considered as a HRV parameter, but it is based, however, on a similar physiological background and can be applicated in comparable ways. Patients need to have ventricular extrasystoles (VES) because HRT is based on the reaction of the system afterward. Healthy individuals without any arrhytmias can not be investigated with HRT. The heart rate directly following a VES increases normally, to decrease a moment later. This pattern is changed or non-existent in patients after myocardial infarction (175). The algorithm returns the parameters turbulence onset and turbulence slope (176).

## Outlook

Since the guidelines of the Task Force were published in 1996, the measurement and calculation of linear parameters have been highly standardized, making investigations comparable and meta-analysis possible. After the introduction of different non-linear parameters, expectations were high, but the role of non-linear algorithms is still unclear after more than two decades. As more than 70 different algorithms have been used (177). Today, HRV beyond its use in pulse watches is still not established in the clinical area although the methods are mature and have been tested extensively. The understanding, however, has increased substantially. Several models have extended its use into psychological and mental health research (55, 178, 179). Finally, 20 years after publishing the first globally used standard of measurement (2), a highly recommendable comprehensive methodological hands-on guideline summarizes state of the art and should be used a new standard (180). In addition, the role of non-linear methods has been recently evaluated and recapitulated (46). Also, a useful guideline to present HRV data has been published (181). HRV as a research and clinical tool is still underrecognized. It should be implemented in several clinical areas within a Bayesian paradigm to improve prediction, diagnosis, and therapy (182).

## Author Contributions

GE has elaborated and written the whole review.

## Conflict of Interest Statement

The author declares that the research was conducted in the absence of any commercial or financial relationships that could be construed as a potential conflict of interest.
